# Case Report: Paraneoplastic melanoma-associated retinopathy mimicking posterior uveitis

**DOI:** 10.3389/fneur.2026.1897579

**Published:** 2026-07-27

**Authors:** Syeda Fatima Abid, Muhammad Hammad Khan, Dina Abdelsalam, Safa Ibrahim, Amy C. Schefler, Andrew G. Lee

**Affiliations:** 1 Department of Ophthalmology, King Edward Medical University, Lahore, Pakistan; 2 Duke Eye Center, Durham, NC, United States; 3 Department of Ophthalmology, Blanton Eye Institute, Houston Methodist Hospital, Houston, TX, United States; 4 Retina Consultants of Texas, Houston, TX, United States; 5 Department of Ophthalmology, Cullen Eye Institute, Baylor College of Medicine, Houston, TX, United States; 6 Departments of Ophthalmology, Neurology, and Neurosurgery, Weill Cornell Medicine, New York, NY, United States; 7 Department of Ophthalmology, University of Texas MD Anderson Cancer Center, Houston, TX, United States; 8 Texas A&M College of Medicine, Bryan, TX, United States; 9 Department of Ophthalmology, The University of Iowa Hospitals and Clinics, Iowa City, IA, United States

**Keywords:** autoimmune retinopathy, melanoma, melanoma-associated retinopathy, posterior uveitis, retinopathy

## Abstract

**Introduction:**

Melanoma-associated retinopathy (MAR) is a rare paraneoplastic ocular manifestation of cutaneous or mucocutaneous melanoma. We describe a novel case of MAR that mimicked an inflammatory or infiltrative retinopathy.

**Patient presentation:**

A 68-year-old female developed bilateral, painless, floaters over six months with accompanying photopsia. Bilateral posterior uveitis was noted, and bilateral vitrectomies were non-diagnostic for malignancy. A systemic evaluation revealed a subcutaneous lesion on the medial aspect of her right thigh. Biopsy of the lesion was consistent with melanoma. The patient was diagnosed with melanoma-associated retinopathy (MAR).

**Conclusion and importance:**

MAR is a vision-threatening paraneoplastic disorder that more commonly arises in patients with an existing diagnosis of melanoma. Most cases of MAR have normal or near normal fundus examinations at onset. Our case is interesting because the fundus presentation mimicked a posterior uveitis and vitrectomy was non-diagnostic. Clinicians should be aware of paraneoplastic MAR and the potential for “masquerade syndrome.” Systemic search for underlying neoplasms including occult melanoma or carcinoma is warranted in such cases.

## Introduction

Melanoma-associated retinopathy (MAR) is a paraneoplastic syndrome that can be difficult to diagnosis. Paraneoplastic ocular syndromes can present with visual loss including MAR, cancer-associated retinopathy (CAR), paraneoplastic uveitis, and paraneoplastic optic neuropathy. In MAR, self-antigens of the eye, expressed by the melanoma, may trigger the production of antiretinal antibodies. This ‘molecular mimicry’ is the presumed underlying mechanism for the development of ocular paraneoplastic syndromes (1). Classic symptoms in MAR include photopsia, nyctalopia, and visual field loss (1). Characteristic electroretinography (ERG) findings (e.g., decreased b-wave) in MAR occur due to bipolar cell dysfunction. Most cases of MAR, however, have a known diagnosis of melanoma. We describe a 71-year-old woman who presented with progressive vision loss in both eyes (OU) and a posterior uveitis OU due to paraneoplastic MAR from an undiagnosed occult non-cutaneous soft tissue melanoma.

### Clinical findings

**Figure 1:**
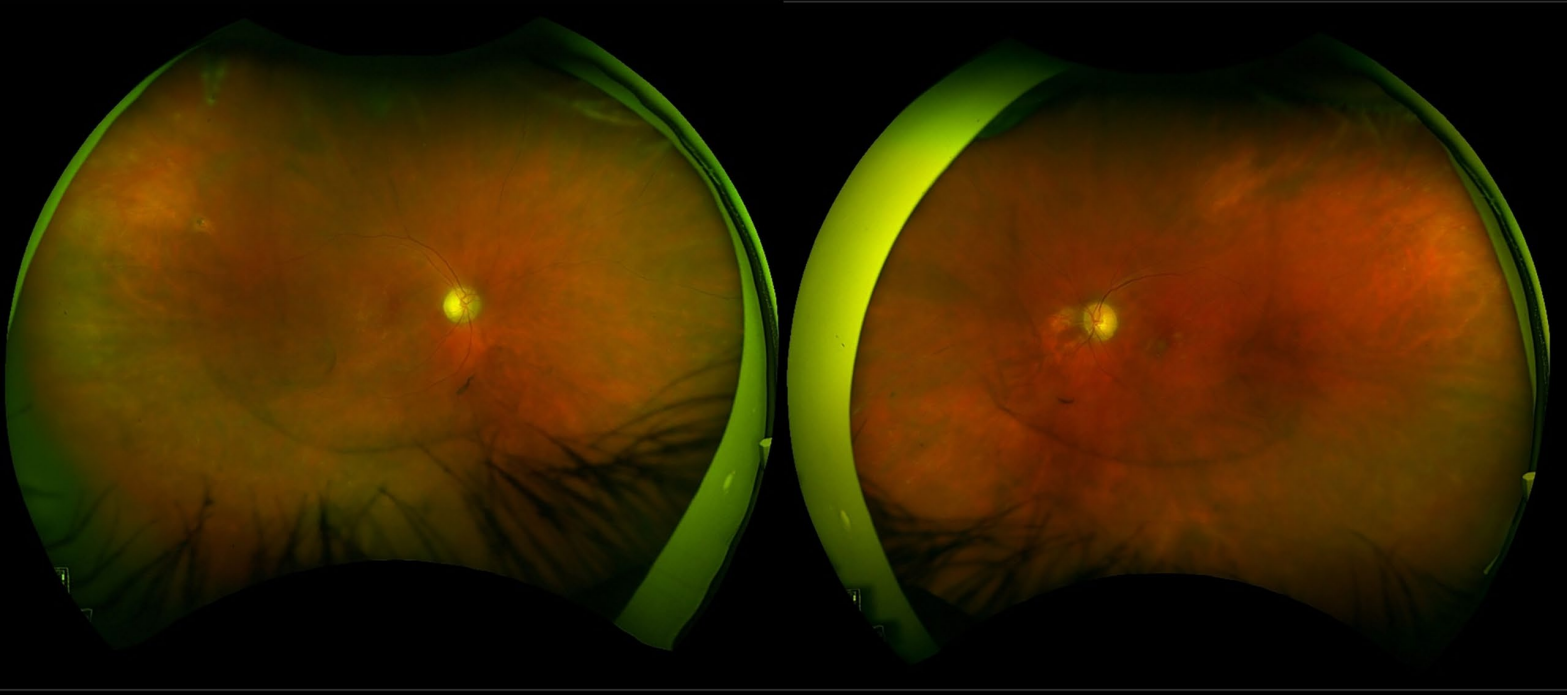
Fundus photograph initial presentation showing dense vitritis in both eyes.

A 71-year-old Caucasian female presented with a six-month history of bilateral simultaneous flashes and floaters. Past medical history included hypertension managed with lisinopril, and hypothyroidism for which she was on replacement levothyroxine. The remainder of the past medical, surgical, social, family, and medication history were non-contributory. She had no known prior cancer or melanoma history. Fundus examination at the initial presentation Sub foveal multifocal yellow deposits with mild RPE mottling (Figure 1).

**Figure 2:**
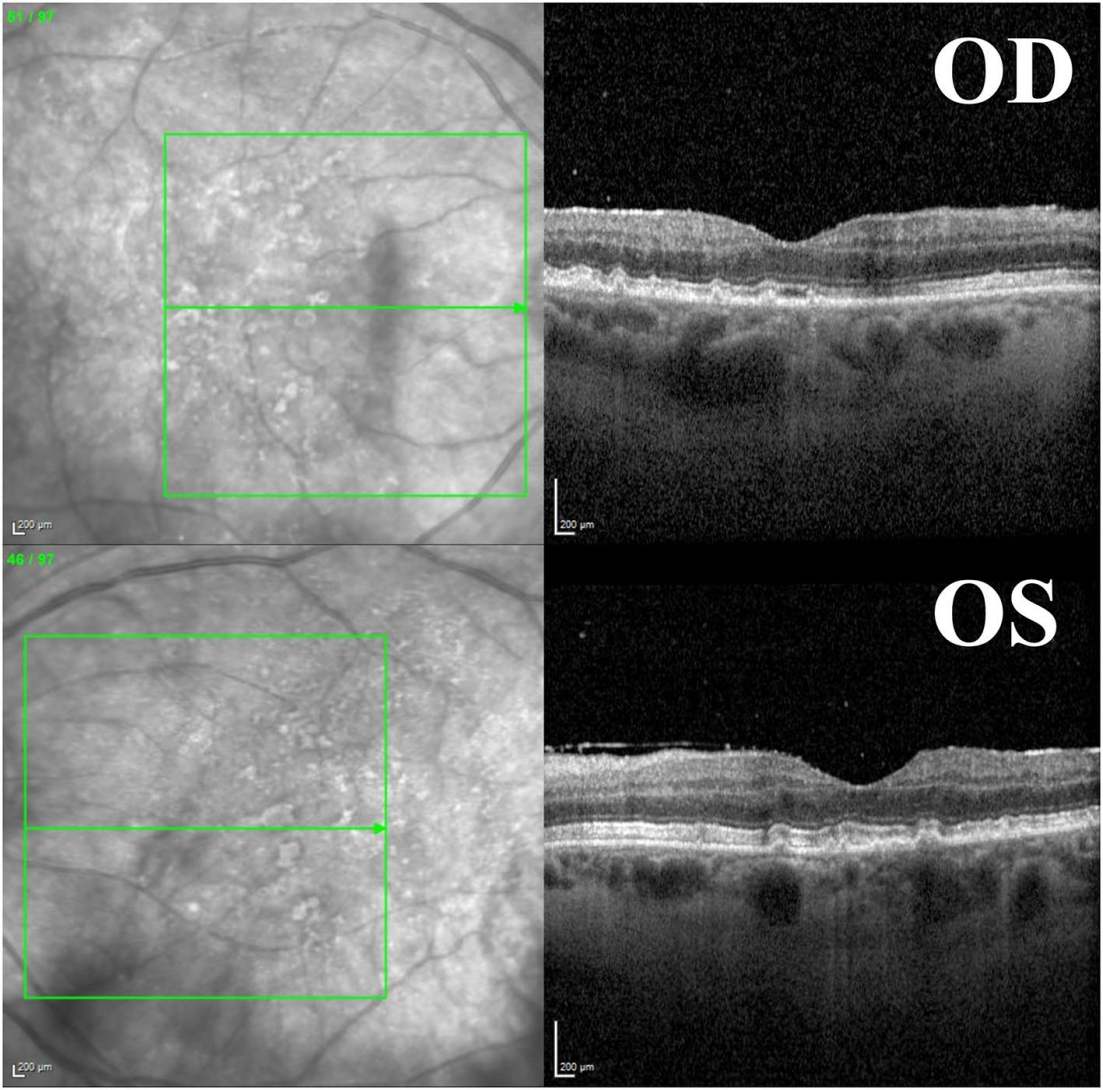
OCT image of both eyes showing vitreous opacities in the vitreous cavity and layered on the hyaloid. There is a preserved foveal depression with sub-RPE deposits and relatively intact inner retinal layers including the ellipsoid zone.

Visual acuity was 20/40 in both eyes. Her intraocular pressure measurements were normal OU. Slit lamp biomicroscopy showed no anterior cells. Ophthalmoscopy demonstrated 3 + vitreous haze and cells and sub-RPE yellow deposits. Optical coherence tomography (OCT) demonstrated numerous vitreous cells deposited on the posterior hyaloid and surface of the internal limiting membrane (ILM) (Figure 2). There was significant optic atrophy in both eyes. B-scan ultrasound demonstrated moderately dense vitreous opacities in both eyes.

Magnetic resonance imaging (MRI) of the brain and orbits with and without contrast was normal. Serologic testing for syphilis, tuberculosis, and Lyme disease was negative. Routine laboratory studies including complete blood count, chemistries, erythrocyte sedimentation rate, and C-reactive protein were normal.

**Figure 3:**
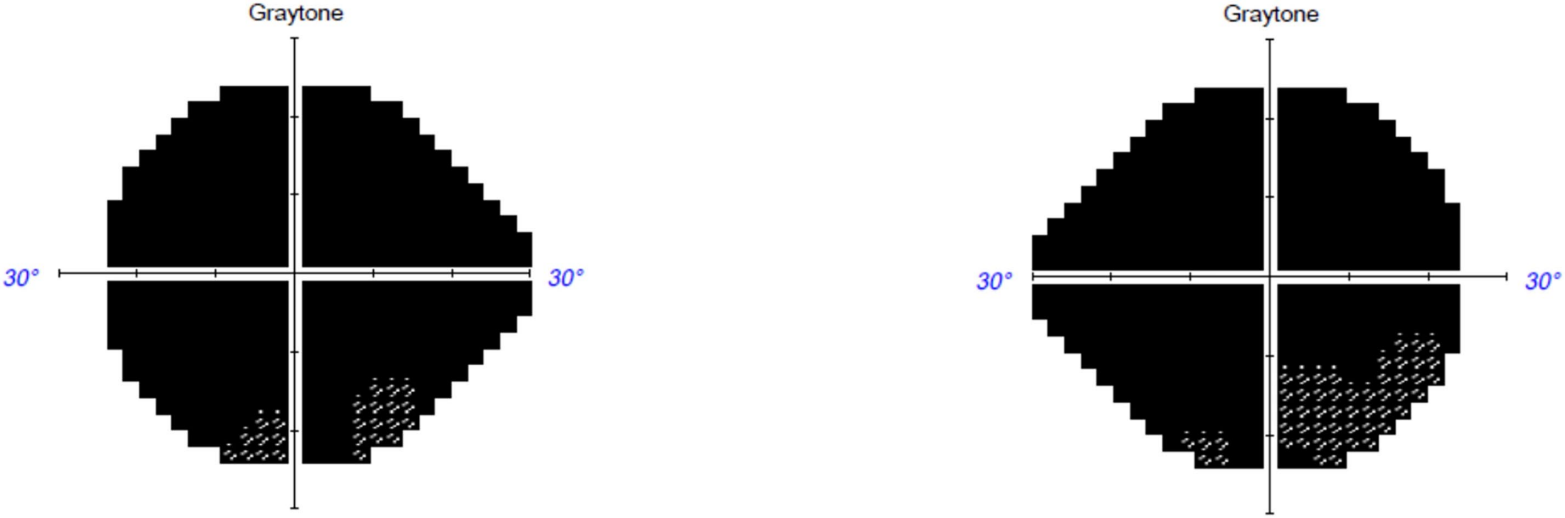
Automated perimetry (Humphrey visual field) showing depressed fields bilaterally.

A diagnostic vitrectomy OS was performed for suspicion of primary vitreoretinal lymphoma (PVRL). Lymphoma. The vitreous pathology showed only polyclonal reactive lymphoplasmacytic infiltrate and granulomatous lesions surrounded by macrophages. Histopathology and immunohistochemistry did not confirm intraocular lymphoma. Diagnostic vitrectomy was then performed in the right eye for the same purpose with identical histopathologic findings. Full field electroretinogram (ERG) showed bilateral moderately attenuated A and B waves OS>OD. Automated perimetry [Humphrey visual field (HVF) 24–1 stimulus was completely depressed OU (Figure 3)].

Physical exam for underlying neoplasm by the primary care physician revealed a solitary, non-painful nodule in the right thigh. In retrospect, the patient reported that the nodule had been present for a year, but it had enlarged more rapidly over the last 6 months. A core needle biopsy showed malignant melanoma. Immunohistochemical stains for HMB45, SOX10, and S100 were consistent with melanoma. The tumor was PRAME+ and BRAF v600E-. Serum paraneoplastic anti-retinal antibodies were positive for aldolase, enolase, transducin-*α*, and TRPM-1.

Serial post-operative examination imaging of the thigh showed regression of the inguinal mass and repeat core biopsies showed only revealed necrotic tissue with a good response to treatment with dual checkpoint inhibitor therapy with ipilimumab, and nivolumab. Staging positron emission tomography (PET) scan of the entire body was negative, and no other lesions were seen. Full dermatologic skin survey showed no melanoma. Gastrointestinal (GI) evaluation showed no GI melanoma.

**Figure 4:**
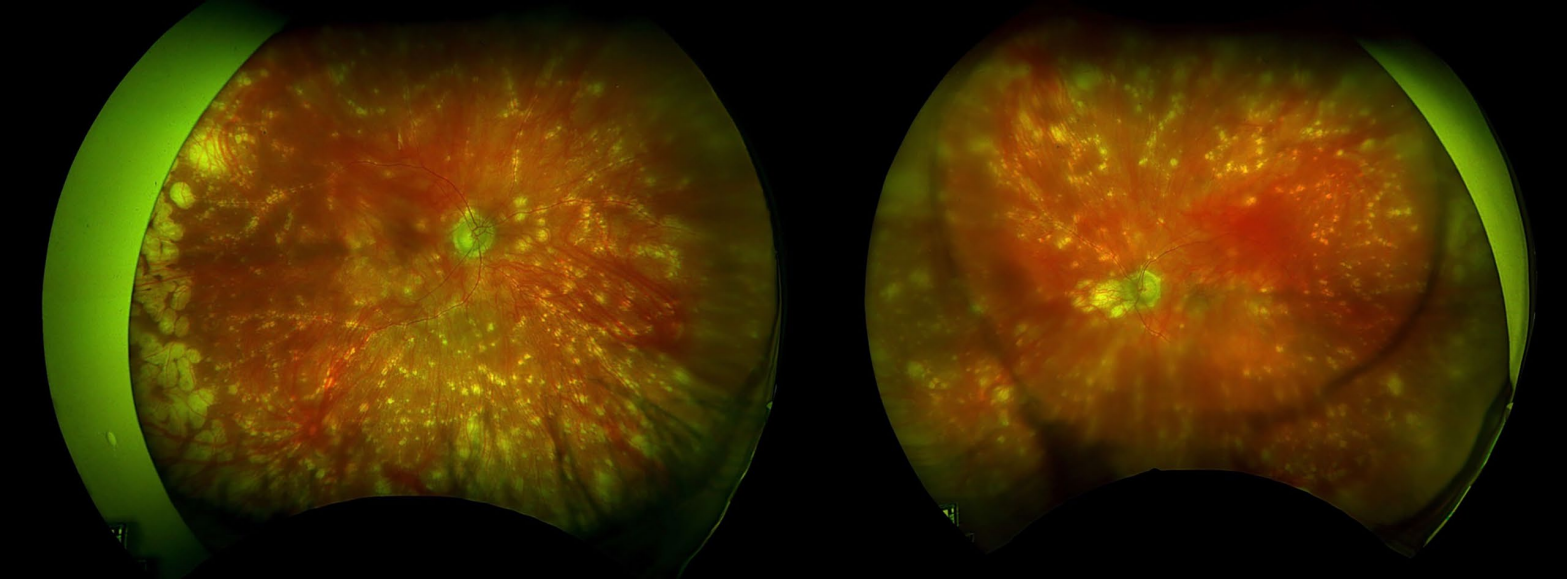
Fundus photograph shows widespread RPE atrophy and optic nerve pallor.

At the last follow up, the patient remains tumor free with no recurrent or metastatic melanoma. No primary cutaneous melanoma was identified. The vision was counting fingers OU and the optic nerve was atrophic OU but the vitreous cell resolved with residual chorioretinal atrophy OU (Figure 4).

## Discussion

Melanoma-associated retinopathy (MAR) is an uncommon paraneoplastic autoimmune syndrome caused by cross-reactivity between retinal antigens and melanoma tumor antigens. MAR typically occurs in patients with a known history of cutaneous or metastatic melanoma, often developing months to years after the primary tumor is diagnosed (2, 3). The average interval between melanoma diagnosis and onset of MAR has been reported as approximately 3–4 years (2). The present case is unique because the presentation was more consistent with posterior uveitis and the ERG findings were not classic. The atypical ERG findings may reflect the patient’s mixed antiretinal antibody profile, with antibodies targeting both bipolar cells and photoreceptor/retinal antigens, producing attenuation of both a- and b-waves rather than a classic MAR pattern. Advanced optic and chorioretinal atrophy may have further contributed to generalized retinal dysfunction. Although the ERG findings overlap with CAR, melanoma was favored given the positive anti-TRPM1, and anti-arrestin. Temporal association with melanoma progression, and absence of another malignancy on PET imaging, dermatologic examination, and gastrointestinal evaluation.

The presumed pathogenesis of MAR involves circulating autoantibodies directed against retinal bipolar cell antigens such as TRPM1, enolase, aldolase, and transducin-*α* (4). These antibodies are thought to arise through molecular mimicry, where melanoma cells aberrantly express retinal antigens, eliciting an immune response that damages the inner retina. Functionally, this leads to dysfunction of the ON-bipolar cells, which manifests as characteristic findings on electroretinography (ERG), a “negative” ERG pattern with preserved a-wave and markedly reduced b-wave amplitude (5).

Classically, MAR typically presents with photopsia, shimmering lights, nyctalopia, and progressive bilateral vision loss, and the fundus findings are minimal or normal OU initially. Over time, patients may develop diffuse retinal pigment epithelium (RPE) abnormalities, optic disc pallor, or vascular attenuation (6). In this patient, posterior vitreous cells and sub-RPE deposits prompted an extensive diagnostic work-up for primary vitreoretinal lymphoma, including a vitrectomy in each eye and imaging, before the remote melanoma lesion was finally identified.

In previously reported cases, ocular symptoms typically emerge after the diagnosis of melanoma or during systemic progression, often signaling metastatic disease (7). This temporal reversal, where the paraneoplastic retinal process precedes the diagnosis of underlying malignancy, underscores the importance of maintaining a high index of suspicion for occult melanoma in patients presenting with unexplained bilateral retinal dysfunction, especially when ERG demonstrates bipolar cell involvement and conventional inflammatory, infectious, and vascular causes have been excluded (Table 1).

**Table 1:** Melanoma-associated retinopathy in patients with a non-cutaneous melanoma, including cases with MAR being diagnosed before melanoma.

Study ID	Patient demographic information	Melanoma location	Time interval between melanoma and MAR diagnosis	Initial ocular presentation	Treatment and/or outcome
Zhou et al. (2025) (10)	66-year-old male	Lower esophagus and cardia	No timeline provided	Bilateral night-time visual impairment for 2 months.	Chemotherapy followed by surgical resection of melanoma; the patients self-reported improved visual function.
Evoy and Lafortune (2020) (11)	63-year-old Male	Conjunctiva	Conjunctival melanoma was noticed 20 years prior, but enlarged over the last 2 years	Right monocular vision loss and phosphenes	Local excision of the tumor with 3-mm margins + 60 mg prednisone and methotrexate; Relapse-free for years
Vercio et al. (2019) (12)	63-year-old female	Left pelvic wall	MAR diagnosis preceded melanoma diagnosis by 2 months.	Sudden, painless onset of night-blindness, and ‘shimmering lights’ in the left eye, over 4 days.	Pembrolizumab for metastatic melanoma; patients reported improvement in vision, with intermittent headaches and fluctuating visual fields.
Rappoport and Leiba (2012) (13)	55-year-old Male	Ileum	Ileal melanoma was diagnosed 3 months after the ocular presentation.	Bilateral depressed vision and photopsia for 1 week.	After ileal resection, the patient’s vision improved substantially. However, he died 3 weeks later from gastrointestinal tumor growth.
Machida et al. (2011) (14)	77-year-old Japanese male	Intranasal melanoma	MAR suspected diagnosis preceded melanoma diagnosis by 6–8 months.	Acute painless loss of night vision in both eyes, with blurred vision and photophobia in the right eye, followed by red-green blindness.	Progressive depression in visual acuity over several months, with no improvement after tumor resection.
Zacks et al. (2001) (15)	69-year-old female	Choroidal melanoma	MAR developed 23 years after the resection of choroidal melanoma in fellow eye, which had been enucleated.	Blurred vision and night blindness for 2 weeks. Serous retinal detachment on slit lamp examination.	Death 11 months after biopsy of lesion.

Interestingly, our patient demonstrated seropositivity for several known MAR-related antibodies, including TRPM-1, aldolase, and enolase, which further confirmed the diagnosis. Following identification of the cutaneous lesion, histopathology revealed PRAME-positive, BRAF-negative melanoma, and treatment with immune checkpoint inhibitors (ipilimumab and nivolumab) was initiated. Systemic and intraocular steroids were not started while PVRL was being ruled out because corticosteroids can suppress lymphoma cells and give a false negative on cytology performed on a vitrectomy specimen. Systemic and intraocular corticosteroids typically are not helpful for MAR and raise the risk of ocular hypertension and rebound inflammation when stopped (8). Interestingly, subsequent ocular examinations showed relative stabilization of retinal findings to retinal and optic atrophy OU, suggesting that systemic immunotherapy may mitigate antibody-mediated retinal injury, as described in isolated reports (9). However, visual recovery in MAR is typically limited, even when tumor control is achieved, due to irreversible retinal damage from prolonged autoimmune activity. Clinicians should be aware that paraneoplastic retinopathy, including MAR, can mimic intraocular infectious, inflammatory, or neoplastic disease. A systematic search including imaging for underlying malignancy should be performed in any patient with suspected paraneoplastic ocular disease.

## Data Availability

The datasets presented in this article are not readily available because of ethical and privacy restrictions. Requests to access the datasets should be directed to the corresponding author.
